# Supplementary material for: In Vivo Imaging and Quantification of Carbon Tracer Dynamics in Nodulated Root Systems of Pea Plants
Source: Plants (Basel). 2022 Feb 25;11(5):632. doi: 10.3390/plants11050632 (PMC8912644; doi:10.3390/plants11050632)
Supplement: Supplementary file 1 [file plants-11-00632-s001.zip › plants-1604522-supplementary.pdf]

Supplemental data

Article title: In vivo imaging and quantification of carbon tracer dynamics in nodulated root systems of pea plants

Authors: Ralf Metzner, Antonia Chlubek, Jonas Bühler, Daniel Pflugfelder, Ulrich Schurr, Gregor Huber, Robert Koller and Siegfried Jahnke

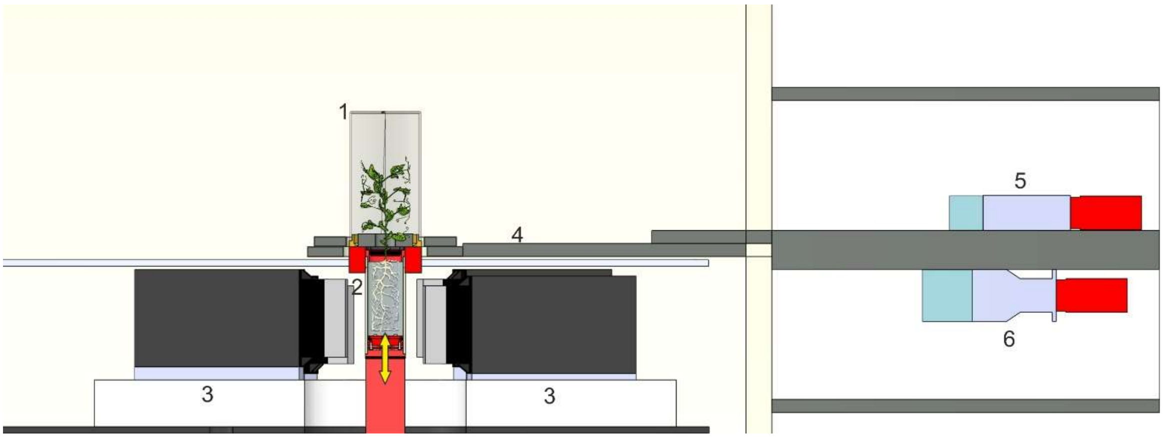

Fig. S1 Set-up of the PET system with auxiliary scintillation detectors. A glass cuvette (1) encloses the aerial part of the plant and is connected to the gas exchange and labelling system. A sealed hydroponics pot (2) contains the belowground part and is aerated with part of the gas flow of the gas exchange system. The pot is placed between the opposing detectors of the PET system PlanTIS (3). The plant is drawn here in the upper of the two measurement positions which were alternated every 5 minutes. In the lower position the pot assembly is 8cm lower as indicated by the yellow arrow. The fields of view of the two additional scintillation detectors for shoot (5) and root (6) are separated by lead shielding (4).

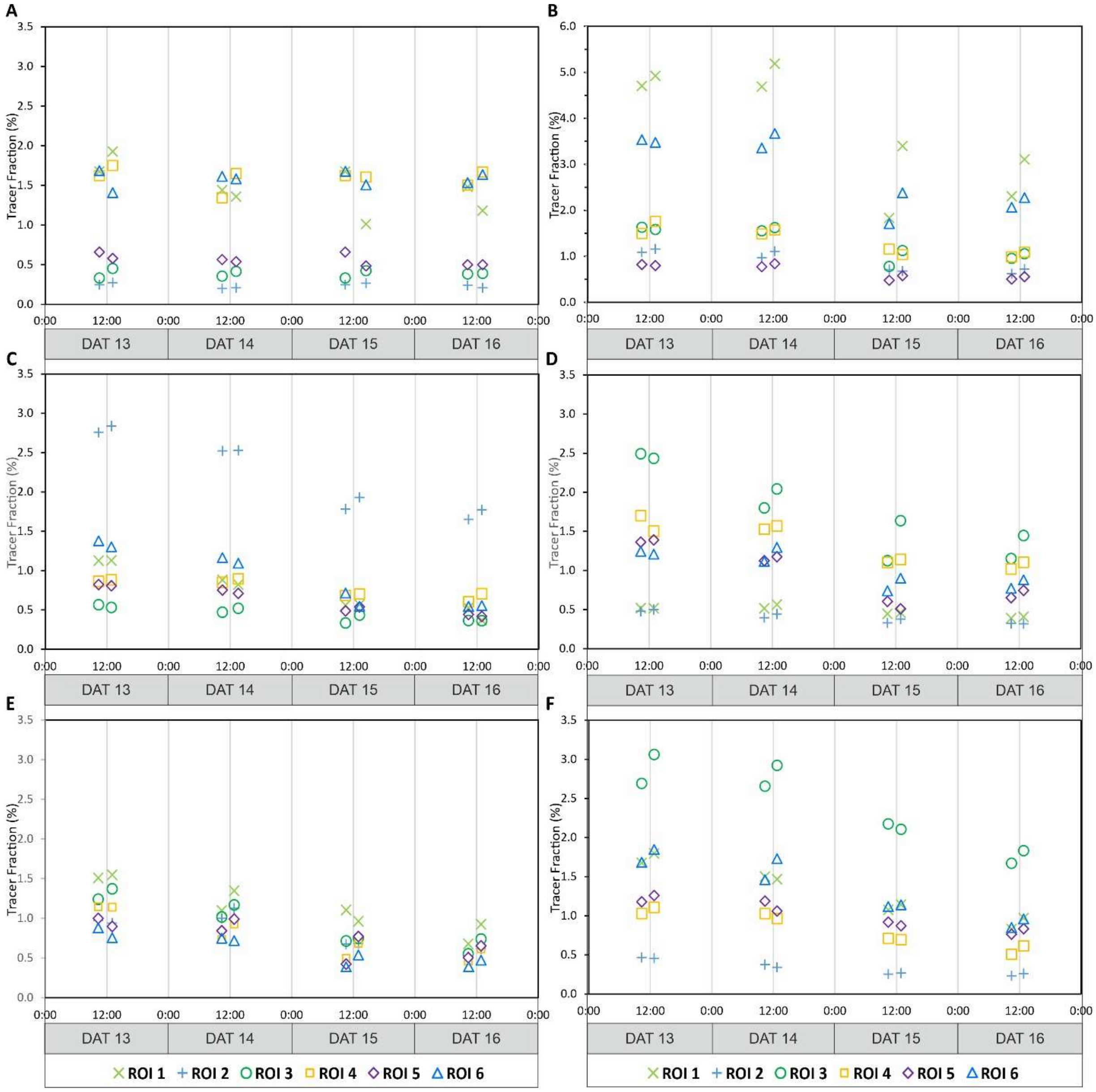

Fig. S2 Carbon tracer allocation to individual nodule ROI of Control and Treatment plants, measured twice on each of four consecutive days. (**a**) Tracer fractions in individual nodules of Control plant 2. (**b**) - (**f**) Tracer fractions in individual nodules of Treatment plants 1 to 5. In each of these, the nutrient solution was exchanged against N+ solution at 16:00 on 14 DAT. Mean tracer fractions were determined as described in Fig. **2**. DAT, days after transfer to pots

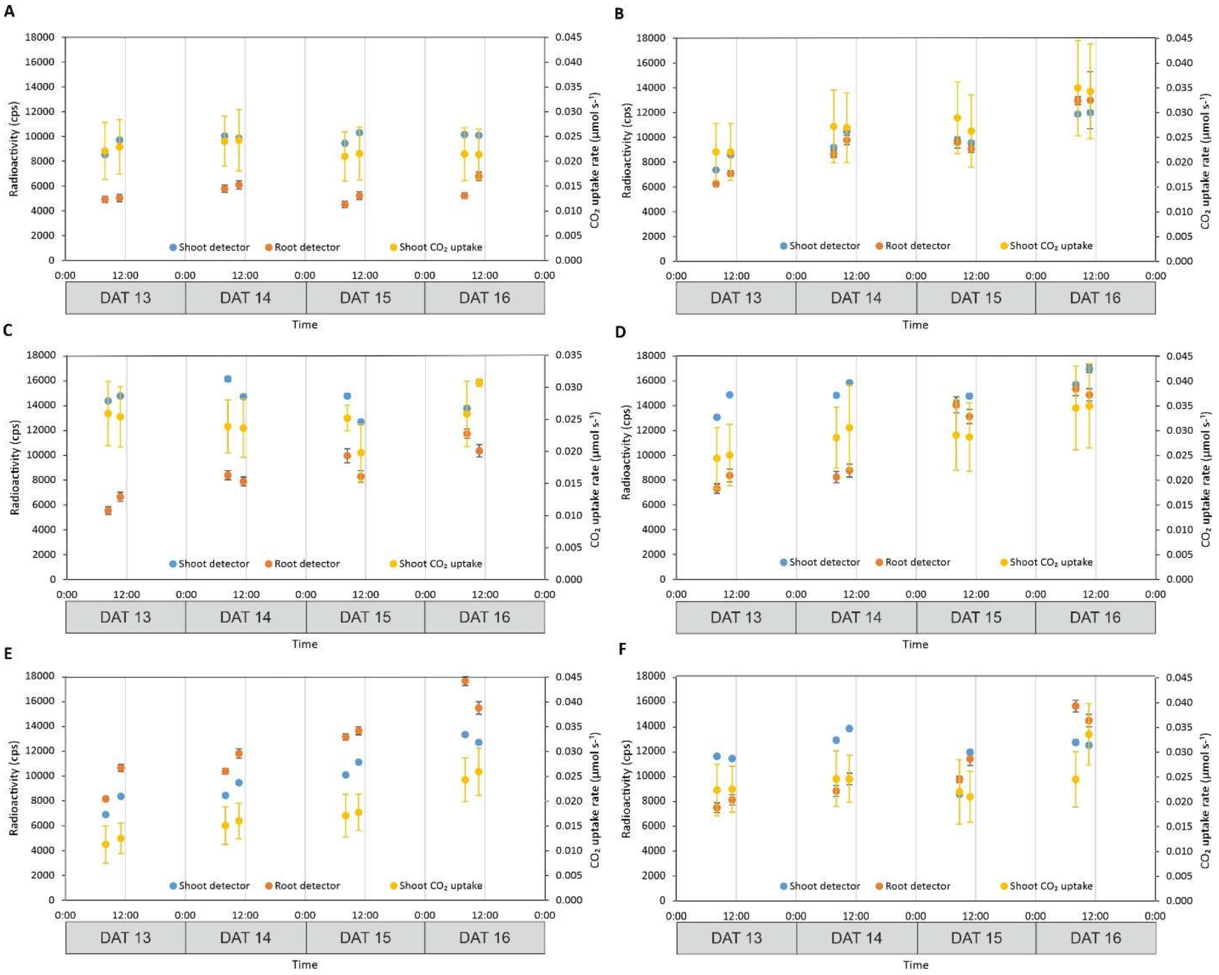

Fig. S3 Whole shoot CO_2 uptake and radioactivity measured by a shoot and a root scintillation detector. For both shoot and root detectors, radioactivity is shown as mean ± SD over the same time span for which mean tracer fractions were calculated from the PET measurements. CO_2 uptake of the whole shoot during the whole time of each measurement is given as mean values ± SD. (**a**) Control plant 2. (**b**) - (**f**) Treatment plants 1-5. DAT, days after transfer to pots

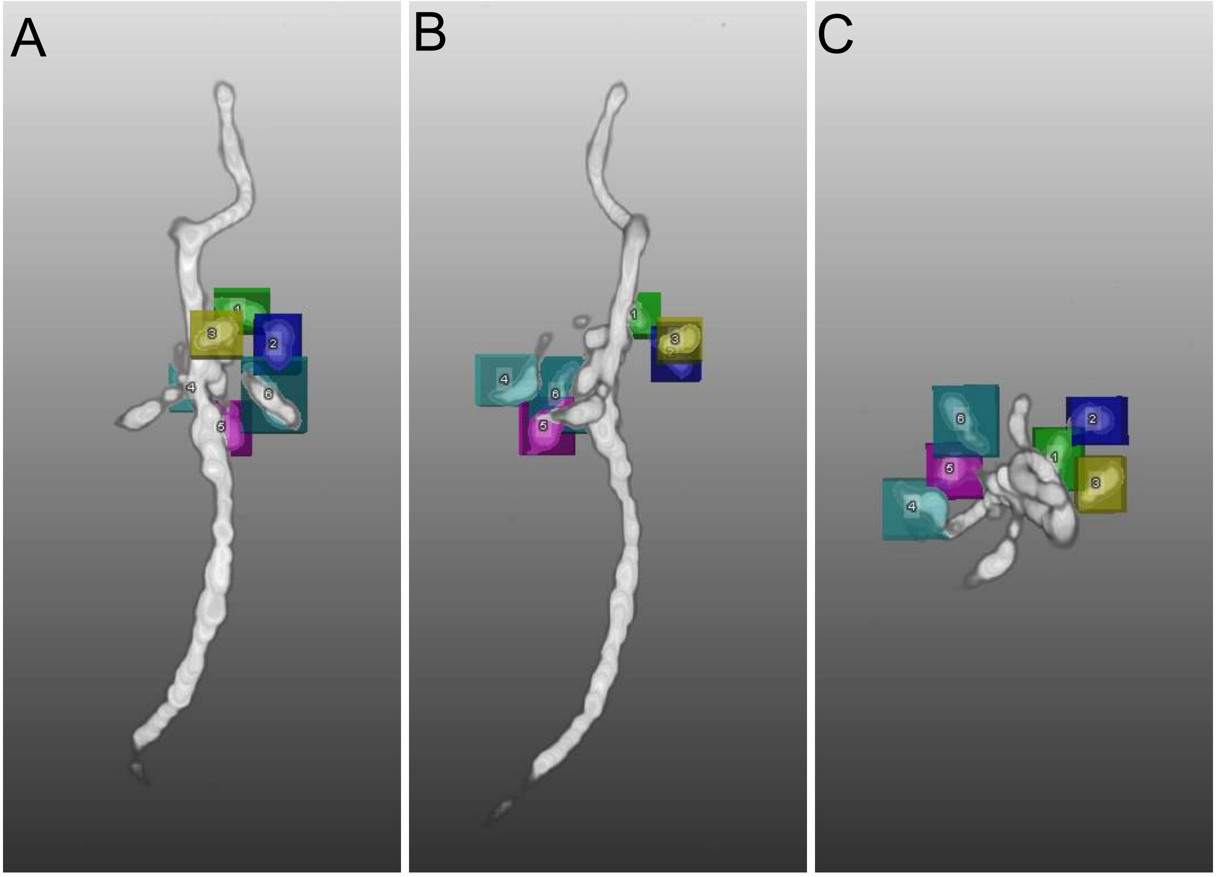

Fig. S4 View of the PET image (grayscale) and ROI depicted in Fig. 4 from three different angles. For technical reasons coloring of the ROI differs slightly from Fig. 4

Table S1 Composition of nutrient solutions for control and treatment with Nitrogen free solution for the control plants and for the treatment plants before start of the treatment labelled N-, the one containing nitrate used N+.

		Salt concentration [mmol l^{-1}]	
		N-	N+
Major nutrient salts	KNO_3	0	5
	KH_2PO_4	0.8	0.8
	$Ca(NO_3)_2$	0	5
	$MgSO_4$	1.0	1.0
	CaCl	2.5	0
	K_2SO_4	3.5	0.8
	NaCl	0.2	0.2
	FeEDTA	0.0563	0.0563
Minor nutrient salts	$CoSO_4$	traces	traces
	H_3BO_3	$3.23\ 10^{-2}$	$3.23\ 10^{-2}$
	$MnSO_4$	$6.49\ 10^{-3}$	$6.49\ 10^{-3}$
	$ZnSO_4$	$7.65\ 10^{-4}$	$7.65\ 10^{-4}$
	$(NH_4)_6O_{24}Mo_7$	$1.46\ 10^{-4}$	$1.46\ 10^{-4}$
	$CuSO_4$	$3.2\ 10^{-4}$	$3.2\ 10^{-4}$

Table S2 p-values for the individual ROI data shown in Fig. S3.

	ROI 1	ROI 2	ROI 3	ROI 4	ROI 5	ROI 6
Control P2	0.124	0.313	0.382	0.357	0.126	0.355
Control P1	0.302	0.079	0.327	0.342	0.264	0.377
Treat P1	0.070	0.046	0.059	0.047	0.054	0.060
Treat P2	0.037	0.039	0.049	0.052	0.037	0.039
Treat P3	0.056	0.041	0.048	0.045	0.052	0.058
Treat P4	0.044	0.059	0.042	0.048	0.073	0.046
Treat P5	0.037	0.040	0.044	0.039	0.040	0.041

p-values were obtained from a t-test with null hypothesis that linear slopes calculated over all 8 measurements per plant were not different from zero. Values below 0.05 highlighted in grey
